# Low triiodothyronine syndrome and selenium deficiency - undervalued players in advanced heart failure? A single center pilot study

**DOI:** 10.1186/s12872-019-1076-5

**Published:** 2019-05-07

**Authors:** Magdalena Fraczek-Jucha, Katarzyna Zbierska-Rubinkiewicz, Małgorzta Kabat, Krzysztof Plens, Radoslaw Rychlak, Jadwiga Nessler, Andrzej Gackowski

**Affiliations:** 1 Department of Coronary Disease and Heart Failure, Jagiellonian University Medical College, Krakow, Poland; 2 Department of Emergency Medical Care, Jagiellonian University Medical College, Krakow, Poland; 3 Department of Coronary Disease and Heart Failure, John Paul II Hospital, Krakow, Poland; 4 Krakow Cardiovascular Research Institute, Krakow, Poland

**Keywords:** Heart failure, Low T3 syndrome, Triiodothyronine, Selenium

## Abstract

**Background:**

The function of deiodinases – selenoproteins converting thyroid hormones may be disturbed by oxidative stress accompanying heart failure. Selenium (Se) may be used by glutathione peroxidase, leading to a lack of deiodinase and triiodothyronine (T3). The aim of the study was the evaluation of the prevalence and clinical significance of low T3 syndrome in heart failure and the assessment of the association of low fT3 and Se deficiency.

**Methods:**

The study group consisted of 59 consecutive patients hospitalized due to decompensated HFrEF NYHA III or IV. Exclusion criteria were: thyroid dysfunction, severe systemic disease, treatment with amiodarone, steroids or propranolol. Group A included 9 patients with low free T3 (fT3) concentration below 3.1 pmol/L. Group B consisted of the remaining 50 patients with normal fT3 levels.

**Results:**

The prevalence of low T3 syndrome was 15.3%. The prevalence of Se deficiency was 74.6%. We demonstrated correlations between fT3 and main clinical variables (i.e. NT-proBNP, LVEF, hsCRP), but we did not find correlation between fT3 and the Se level. Kaplan-Meier survival analysis showed lower survival probability in patients with low fT3 (*p* < 0.001).

**Conclusions:**

Low T3 syndrome is frequently found in patients with HFrEF and is associated with a poor outcome. We did not identify any significant correlation between Se and fT3 level.

## Background

It has been proven that either hyperthyroidism or hypothyroidism has a harmful influence on the cardiovascular system and can lead to heart failure (HF) [1]. However, advanced HF is a complex multiorgan syndrome leading to many hormonal and immunologic alterations including low triiodothyronine (T3) syndrome characterized by low triiodothyronine, elevated or normal reverse triiodothyronine (rT3) and normal or slightly disturbed thyroid stimulating hormone (TSH) and tetraiodothyronine (T4) levels [1–3]. T3 is the active hormone in peripheral cells, while T4 is a prohormone [4, 5]. HF may be associated with lowered T3 concentration in 24.5% of HF patients [6]. Several mechanisms may cause disturbances of T4 to T3 conversion. One of them may be decreased activity of deiodinase 1 (D1) and increased expression and activity of deiodinase 3 (D3). Increased expression of the D3 gene (*DIO3*) may occur due to hypoxia and inflammation [7, 8].

There is also a hypothesis that the function of deiodinases may be disturbed by oxidative stress and selenium (Se) deficiency accompanying HF. The role of oxidative stress in the onset and development of cardiovascular diseases is widely described. Heart failure may lead to an imbalance between pro-oxidant and anti-oxidant enzymes leading to an excess of reactive oxidant species (ROS). There is growing evidence that ROS may lead to nitric oxide synthase uncoupling, lipid peroxidation, endothelial dysfunction, vascular smooth muscle growth, inflammatory molecules enhancement, DNA oxidative damage and platelet activation [9]. The above may contribute to the progression of HF.

Glutathione peroxidase (GPX) may be considered an oxidative stress defense marker. Se levels correlate with GPX enzymatic activity [10, 11]. Deiodinases and GPX are selenoproteins competing for Se [5]. This is why Se deficiency may lead to both GPX deficiency and impaired T4 to T3 conversion. Both phenomena may be caused by advanced HF. On the other hand, oxidative stress and low T3 syndrome may contribute to HF progression [11].

Low T3 syndrome accompanying HF can cause many disturbances. Lack of thyroid hormones (TH) may cause downregulation of the myosin heavy chain α (α-MHC) gene expression *(MYH6),* leading to a deterioration of systolic function of the heart. TH deficiency contributes to a decrease in sarcoplasmic/endoplasmic reticulum calcium ATPase2 (SERCa2) by downregulation of the *ATP2A2* gene. The increase in its inhibitor – fosfolamban (PLN), caused by the *PLN* gene upregulation, may decrease calcium reuptake during diastole, causing myocardial relaxation impairment. TH activate phosphatidylinositol 3-kinase (PI3K) and serine/threonine-protein kinase (AKT) signaling pathways by nongenomic action, inducing production of endothelial nitric oxide [1, 12, 13]. In addition, TH (especially T3) have a direct concentration-dependent vasodilatory effect [14]. Also, a low level of TH may affect the function of ion channels, leading to arrhythmia [1, 12, 13]. TH deficiency may affect cardiac mitochondrial biogenesis [15]. Many of the above mechanisms may potentially worsen the clinical course of HF. However, the clinical significance of low T3 syndrome is poorly studied.

A few studies suggest maladaptive character of low T3 syndrome. One study showed that low T3 syndrome is a prognostic predictor of death in patients with heart diseases [16]. A few studies suggest that low T3 levels are associated with HF severity and are more prevalent in NYHA class III-IV [16]. A low T3 level was shown to be a predictor of prolonged hospital stay [17], all-cause and cardiac mortality in HF [18–20]. Several studies showed that low fT3 concentration may have a similar prognostic value as NT-proBNP in chronic and acute HF [21–24]. However, the coincidence of low T3 syndrome and selenium deficit was not tested in HF patients. The aim of the study was the evaluation of the prevalence and clinical significance of low T3 syndrome in decompensated HF and the relation of low fT3 to selenium deficiency.

## Methods

### Study population

The study protocol of this prospective cohort study was approved by the Ethics Committee. All procedures performed in studies involving human participants were in accordance with the ethical standards of the Helsinki Declaration. From June of 2015 to August of 2017 we prospectively evaluated 59 consecutively hospitalized patients who gave written informed consent and fulfilled the inclusion criteria: decompensated heart failure with reduced ejection fraction (HFrEF), NYHA class III or IV. The diagnosis was made according to the ESC Guidelines for the diagnosis and treatment of acute and chronic heart failure [25]. Exclusion criteria were: admission due to acute coronary syndrome, previous or current thyroid disease (abnormalities in thyroid physical examination, abnormal thyroid morphology ascertained by previous imaging tests, abnormal serum level of TSH at admission, subclinical or overt hyperthyroidism, subclinical or overt hypothyroidism, treatment with amiodarone, glucocorticosteroids and/or propranolol), clinical evidence of severe systemic disease (e.g., inflammatory or autoimmune disease, neoplasm, chronic renal disease (GFR < 30 ml/min/1,73m^2^). All the patients received optimal medical treatment. Depending on fT3 concentration, 2 study groups were distinguished: **Group A** consisting of patients with a fT3 concentration below the normal limit and **Group B** with normal fT3 plasma levels.

### Biochemical tests

Serial blood samples were collected to assess the thyroid profile, as well as to carry out biochemical and hematology testing. On the 1st and 3rd day of hospitalization and on the follow-up visit the patients had laboratory tests such as TSH [electrochemiluminescent immunoassay (ECLIA) method, sandwich test], free tetraiodothyronine (fT4), free triiodothyronine (fT3) [electrochemiluminescent immunoassay (ECLIA) method, competitive test], rT3 [radioisotope method] was determined on the 3rd day of hospitalization.

Reference values in our laboratory were: TSH (0.27–4.2 μIU/ml), fT3 (3.10–6.80 pmol/L), fT4 (12.0–22.0 pmol/L), rT3 (0,09–0,35 ng/ml).

NT-proBNP [electrochemiluminescent immunoassay (ECLIA) method, sandwich test] levels were analyzed on the 1st and 3rd day of hospitalization and on the follow-up visit. The reference value for NT-proBNP in our laboratory is < 125.0 pg/ml. Serum markers of inflammatory state: hsCRP [immunoturbodimetric method] and white blood count (WBC) [Hydro Dynamic Focusing flow cytometry method] were performed on the 1st and 3rd day of hospitalization. Creatinine levels [Jaffé Gen.2 method, rate blanked, compensated] and eGFR/CKD-EPI/ were assessed at admission.

Samples for serum selenium level were taken on the 3rd day of hospitalization using the Vacutainer system. After collection, blood was left to clot for at least 30 min. Before 120 min after collection, the samples were centrifuged for serum separation (1300G, 12 min). After that, the serum samples were frozen at − 80 C. On the day of analysis, samples were thawed and centrifuged (5000G, 5 min). Inductively coupled plasma mass spectrometry (ICP-MS) was used for analyses. Selenium determination was performed on the ICP mass spectrometer NexION 350D (PerkinElmer, Shelton, USA). The spectrometer was equipped with dynamic reaction cell operating with high-purity methane. It was calibrated using an external calibration technique. Calibration standards were prepared from 10 μg/ml Multi-Element Calibration Standard 3 (PerkinElmer, Shelton, USA) by diluting with to the final concentration of 0,1; 0,5; 1,0; 2,0; 5,0; 10 μg/l. Correlation coefficients for calibration curves were always greater than 0,999. An analysis protocol assumed 100-fold dilution of serum in blank reagent. Blank reagent consists of 10 ml of 65% Suprapur Grade nitric acid (Merck, Germany), 0,20 ml of Triton X-100 (PerkinElmer, USA) filled to the mark of 1 l flask with class I deionized water (Merck Millipore). The Germanium isotope (Ge^74^) was set as the internal standard.

Reference values of selenium in the local laboratory were: females < 50 years old (75.0–85.0 μg/l), females > 50 years old (65.0–75.0 μg/l), males < 60 years old (85.0–95.0 μg/l), males > 60 years old (70.0–90.0 μg/l).

### Echocardiographic assessment

The transthoracic echocardiographic evaluation of the left ventricle was performed during hospitalization and on the follow-up visit (Philips IE 33, Amsterdam, The Netherlands) by the same echocardiographer. Afterwards, records were reviewed and confirmed blindly by another echocardiographer. The following parameters were analyzed: dimensions, volumes and sphericity of the left ventricle (LVEDD, LVESD, LVEDV, LVESV, SI), left ventricle ejection fraction (LVEF), the rate of LV systolic pressure rise (+dp/dt), left ventricle filling profile, E/E’ index (E’ was measured on medial and lateral aspect of mitral annulus and averaged), mitral annular plane systolic excursion (MAPSE), tricuspid annular plane systolic excursion (TAPSE) and right ventricle systolic pressure (RVSP). The incidence of moderate or severe functional mitral valve regurgitation was assessed according to EACVI guidelines [26].

### 24-h Holter ECG monitoring

24-h Holter ECG monitoring was performed during hospitalization (Reynolds Pathfinder system, Snoqualmie, USA). Maximal, minimal and mean sinus rhythm frequency, as well as maximal and minimal ventricular rhythm in case of atrial fibrillation or flutter were calculated during hospitalization. In addition, heart rate variability for sinus rhythm (HRV), severe bradycardia, atrioventricular conduction disturbances and occurrence of ventricular or supraventricular arrhythmias were evaluated.

### Study end-points

Major adverse events (MAE) included: resuscitated cardiac arrest, cardiovascular death, re-hospitalization for decompensated heart failure requiring intravenous diuretics and/or catecholamines administration, evidence of ventricular tachycardia, severe sinus bradycardia or atrioventricular blocks. We also analyzed the occurrence of the composite endpoint of the above adverse events and the duration of hospital stay. Each of the events was analyzed during the hospital stay and during the follow-up of 137.53 (±36.49) days. All patients entered the survival analysis, including 43 participants attending the control ambulatory visit (3 - group A, 40 – group B), and 12 participants were contacted by phone (2 - group A, 10 – group B). Four participants from group A died before the scheduled follow-up visit.

## Statistical analysis

Categorical variables are presented as numbers and percentages. Continuous variables are expressed as mean ± standard deviation (SD) or median and interquartile range (IQR). We assessed the normality using the Shapiro-Wilk test. For the equality of variances, we used the Levene’s test. Differences between groups were compared using the Student’s or Welch’s t-test depending on the equality of variances for normally distributed variables. We used the Mann-Whitney U-test for non-normally distributed continuous variables. Categorical variables were compared by the Pearson’s chi-squared test or the Fisher’s exact test if 20% of cells had an expected count of less than 5. We used the Pearson’s test or the Spearman rank correlation test (depending on normality) to calculate the coefficient correlation measuring dependence. The univariate Cox proportional hazard analysis was performed to identify predictors of the outcomes. Receiver operating characteristic (ROC) curves were analyzed to determine the optimal cut-off values for parameters. We drew Kaplan-Meier curves to analyze an event-free survival in selected risk groups. The log-rank statistic was used to test the differences in the outcomes between the groups. We performed the statistical analyses with JMP®, Version 14.0.0 (SAS Institute INC., Cary, NC, USA) and using R, Version 3.4.1 (R Core Team. R: A language and environment for statistical computing. R Foundation for Statistical Computing. Vienna, Austria, 2017. https://www.r-project.org/). *P*-value less than 0.05 was considered as statistically significant.

## Results

Baseline characteristics of the study group are shown in Table 1. The prevalence of low T3 syndrome was 15.3% (*n* = 9) while the prevalence of selenium deficiency was 74.6% (*n* = 44).

**Table 1:** Baseline characteristics of study population according to fT3 concentration

Characteristics	Group A Low fT3 < 3.10 pmol/L (*n* = 9)	Group B Normal fT3 ≥ 3.10 pmol/L (*n* = 50)	*p*-value
Age, years	65.0 (58.0–78.5)	64.5 (58.5–71.5)	0.4047
Male sex, n (%)	7 (77.8%)	41 (82.0%)	0.6697
Body mass index (kg/m^2^)	20.8 (19.4–26.4)	30.1 (26.9–33.3)	0.0008
Main etiology of heart failure			
Ischemic	4 (44.4%)	25 (50.0%)	0.7117
Valvular	2 (22.2%)	6 (12.0%)	
Idiopathic/Other	3 (33.3%)	19 (38.0%)	
Medical history, n (%)			
Hypertension	8 (88.9%)	42 (84.0%)	1.0000
Dyslipidemia	7 (77.8%)	44 (88.0%)	0.5946
Type 2 diabetes mellitus	3 (33.3%)	23 (46.0%)	0.7176
Atrial fibrillation	4 (44.4%)	28 (56.0%)	0.7187
Smoking	3 (33.3%)	18 (36.0%)	1.0000
Medications prior to admission, *n* (%)			
Beta blockers	8 (88.9%)	46 (92.0%)	0.5768
ACEI	4 (44.4%)	38 (76.0%)	0.1033
ARB	0 (0.0%)	3 (6.0%)	1.0000
MRA	6 (66.7%)	34 (68.0%)	1.0000
Diuretics	7 (77.8%)	45 (90.0%)	0.2880
Ivabradine	3 (33.3%)	4 (8.0%)	0.0644
Digoxin	9 (18.0%)	2 (22.2%)	0.6697
Electrotherapy prior to admission, n (%)			
ICD	3 (33.3%)	14 (28.0%)	0.7080
CRT	2 (22.2%)	5 (10.0%)	0.2880
Pacemaker	1 (11.1%)	3 (6.0%)	0.4940
iv medication during hospitalization			
Iv diuretics	8 (88.9%)	31 (72.1%)	0.4203
Iv inotropic drugs	6 (66.7%)	4 (8.0%)	0.0003
Initial clinical findings			
Rales, n (%)	7 (77.8%)	22 (44.0%)	0.0797
Peripheral edema, n (%)	8 (88.9%)	23 (46.0%)	0.0274
Heart rate (beats/min)	88.0 (72.5–115.0)	80.0 (70.0–101.0)	0.4400
Systolic blood pressure (mmHg)	108.7 (±14.9)	129.0 (±22.8)	0.0128
Diastolic blood pressure (mmHg)	73.0 (±11.17)	82.3 (±15.2)	0.0876
NYHA class			
III, n (%)	3 (33.3%)	34 (68.0%)	0.0661
IV, n (%)	6 (66.7%)	16 (32.0%)	

We found no difference between groups regarding age, sex, HF etiology, comorbidities, pharmacotherapy prior to admission, use of electrotherapy and NYHA class. Patients with low T3 syndrome had a lower body mass index and systolic blood pressure. They also more frequently suffered from peripheral edema and required inotropic drugs during their hospitalization.

The repeated analysis of the hormone levels showed that 2 patients with initially low T3 syndrome became euthyroid, whereas 1 previously euthyroid patient developed low T3 syndrome. Additionally, 1 previously euthyroid patient developed hyperthyroidism.

Laboratory and echocardiographic data are presented in Table 2 and Table 3.

**Table 2:** Laboratory data according to serum fT3 concentration

Characteristics	Group ALow fT3 < 3.10 pmol/L (*n* = 9)	Group B Normal fT3 ≥ 3.10 pmol/L (*n* = 50)	*p*-value
TSH (1 day), μIU/ml	2.3 (0.98–3.0)	1.5 (0.9–1.9)	0.1984
TSH (3 day), μIU/ml	2.2 (1.3–3.0)	1.5 (1.2–2.0)	0.1049
fT3 (1 day), pmol/l	2.7 (±0.7)	4.4 (±0.7)	<0.0001
fT3 (3 day), pmol/l	2.6 (±0.8)	4.3 (±0.6)	<0.0001
fT4 (1 day), pmol/l	16.9 (±2.9)	18.1 (±3.0)	0.2455
fT4 (3 day), pmol/l	16.5 (±3.3)	17.6 (±2.4)	0.2803
fT3/fT4 ratio (1 day)	0.16 (±0.04)	0.25 (±0.05)	<0.0001
fT3/fT4 ratio (3 day)	0.16 (±0.06)	0.25 (±0.04)	<0.0001
rT3, ng/ml	0.18 (±0.09)	0.22 (±0.09)	0.1854
NT-proBNP (1 day), pg/ml	7330.0 (5243.0–28,033.0)	2714.5 (1762.3–5968.3)	0.0016
NT-proBNP (3 day), pg/ml	6909.0 (5143.0–21,934.5)	2027. (790.5–3378.6)	<0.0001
hsCRP (1 day), mg/l	10.2 (4.5–37.0)	4.1 (1.8–9.3)	0.0369
hsCRP (3 day), mg/l	21.0 (3.6–46.0)	5.1 (1.5–8.6)	0.0269
WBC (1 day), 10^3	7.3 (5.5–9.7)	7.9 (6.3–9.3)	0.5692
WBC (3 day), 10^3	7.6 (5.4–10.6)	7.1 (6.1–7.7)	0.4932
Selenium, μg/l	58.2 (31.7–71.0)	59.7 (47.9–71.2)	0.4735
Creatinine (1 day), μmol/l	109.0 (77.5–121.5)	103.0 (81.5–123.0)	0.8720
eGFR/CKD-EPI/ (1 day)	66.0 (52.0–81.0)	64.0 (50.0–84.0)	0.7553

**Table 3:** Echocardiographic data of study population according to fT3 concentration

Characteristics	Group A Low fT3 < 3.10 pml/L (*n* = 9)	Group B Normal fT3 ≥3.10 pmol/L (*n* = 50)	*p*-value
LVEDD, mm	65.5 (±12.7)	65.6 (±9.9)	0.9483
LVESD, mm	59.0 (±14.0)	55.9 (±11.4)	0.4692
LVEDV, ml	212.9 (±83.4)	238.4 (±86.8)	0.4187
LVESV, ml	178.8 (±76.3)	184.6 (±78.9)	0.8386
SI	0.6 (±0.07)	0.64 (±0.12)	0.3395
LVEF, %	16.1 (±5.7)	25.4 (±7.4)	0.0008
MAPSE, mm	4.0 (3.6–4.8)	7.0 (5.0–8.9)	0.0051
E/A	2.7 (2.2–3.2)	1.3 (0.8–2.2)	0.0082
Number of pts. with restrictive mitral filling pattern, n (%)	5 (8.3%)	13 (46.4%)	0.1801
E/E’	15.2 (±5.6)	17.5 (±8.5)	0.4321
RVSP, mmHg	44.0 (39.0–62.5)	35.0 (31.0–48.5)	0.0794
TAPSE, mm	12.8 (±4.8)	15.8 (±5.0)	0.1066
+dP/dt, mmHg/s	466.0 (404.5–824.5)	606.0 (457.0–757.0)	0.4222
Functional mitral regurgitation, n (%)	5 (55.6%)	21 (42.0%)	0.4885

Patients with low fT3 presented: lower fT3/fT4 ratio, higher levels of NT-proBNP and hsCRP. There was a non-significant tendency for lower Se levels in the low fT3 group. In the low fT3 group we observed a higher mitral inflow, E/A ratio but lower LVEF and MAPSE.

On the follow-up visit laboratory tests and echocardiography were performed but results were not analyzed because of a limited number of survivors in group A (4 patients died before the scheduled visit).

We found no differences between the low fT3 group and normal fT3 group in parameters analyzed during 24-h ECG monitoring: max. HR (medians, 95.0 [88.5–106.5] vs. 99.0 [87.0–116.3], *p* = 0.5477), min. HR (60.3 ± 10.6 vs. 63.3 ± 11.1, *p* = 0.4634), med. HR (74.2 ± 1. vs. 77.2 ± 13.6, *p* = 0.5409), HRV (*p* = 0.8897), number of premature ventricular contractions (medians, 2887.0 [416.5–10,197.5] vs. 871.5 [137.3–3055.5], *p* = 0.1912), occurrence of non-sustained ventricular tachycardia (6 [66.7%] vs. 34 [68.0%], *p* = 1.0), occurrence of sustained ventricular tachycardia (0.0% vs. 0.0%), number of premature supraventricular contractions (medians, 0.0 [0.0–288.0] vs. 3.50 [0.0–163.3], *p* = 0.9552), occurrence of supraventricular tachycardia (2 [22.2%] vs. 19 [38.0%], *p* = 0.4687), occurrence of severe bradycardia (0.0% vs. 0.0%) and occurrence of atrioventricular block type II Mobitz II or type III (0 [0.0%] vs. 1 [2.0%], *p* = 1).

Correlations between fT3 concentration, fT3/fT4 ratio and main clinical variables are shown in Table 4.

**Table 4:** Correlation analysis

Variable	By variable	Correlation	*p*-value
fT3	hsCRP	−0.4505	0.0003
fT3	selenium	0.2265	0.0846
fT3	NT-proBNP	−0.5368	<0.0001
fT3	LVEF	0.4311	0.0007
fT3	MAPSE	0.2819	0.0305
fT3	+dp/dt	0.2981	0.0467
fT3	TAPSE	0.3345	0.0096
fT3	E/A	−0.4496	0.0087
fT3/fT4	hsCRP	−0.4855	<0.0001
fT3/fT4	selenium	0.1139	0.3906
fT3/fT4	NT-proBNP	−0.5408	<0.0001
fT3/fT4	LVEF	0.4865	<0.0001
fT3/fT4	MAPSE	0.3341	0.0097
fT3/fT4	+dp/dt	0.4831	0.0079
fT3/fT4	TAPSE	0.2837	0.0295
fT3/fT4	E/A	−0.4552	0.0078

The hospital stay in the low fT3 group was longer in comparison to the normal fT3 group (medians, 18.0 [14.5–36.5] vs. 5.0 [4.0–8.3], p = < 0.0001). Endpoints were more frequent in the low fT3 group: in-hospital mortality (33% vs. 0.0%, *p* = 0.0026), resuscitated cardiac arrest (2% vs 33.3%, *p* = 0.0095), cardiovascular death (44.4% vs. 0.0%, *p* = 0.0003).

A univariate analysis revealed that fT3 and fT3/fT4 ratio were predictors of survival (Table 5). Unfortunately, a multivariate analysis was not possible due to a limited sample size.

**Table 5:** Univariate analysis of predictors for cardio-vascular mortality

Univariate			
Variable	HR per	HR (95% CI)	*p*-value
fT3 (1 day), pmol/l	1	0.230 (0.060–0.649)	0.0051
fT3 (3 day), pmol/l	1	0.249 (0.090–0.619)	0.0036
fT3/fT4 ratio (1 day)	0.1	0.079 (0.006–0.560)	0.0101
fT3/fT4 ratio (3 day)	0.1	0.073 (0.007–0.436)	0.0038
NT-proBNP (1 day), pg/ml	1000	1.102 (1.012–1.197)	0.0290
NT-proBNP (3 day), pg/ml	1000	1.144 (1.043–1.257)	0.0074
LVEF, %	1	0.768 (0.577–0.929)	0.0033

**Fig. 1:**
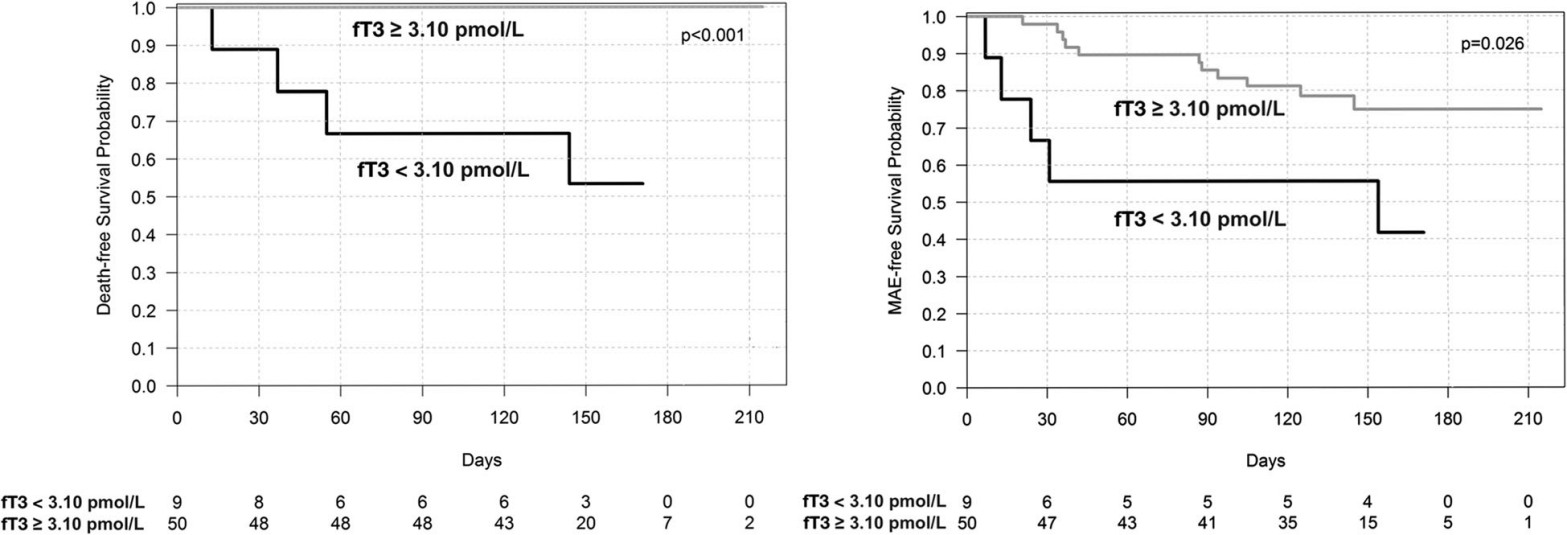
Kaplan-Meier survival curves for patients with low fT3 vs. normal fT3

**Fig. 2:**
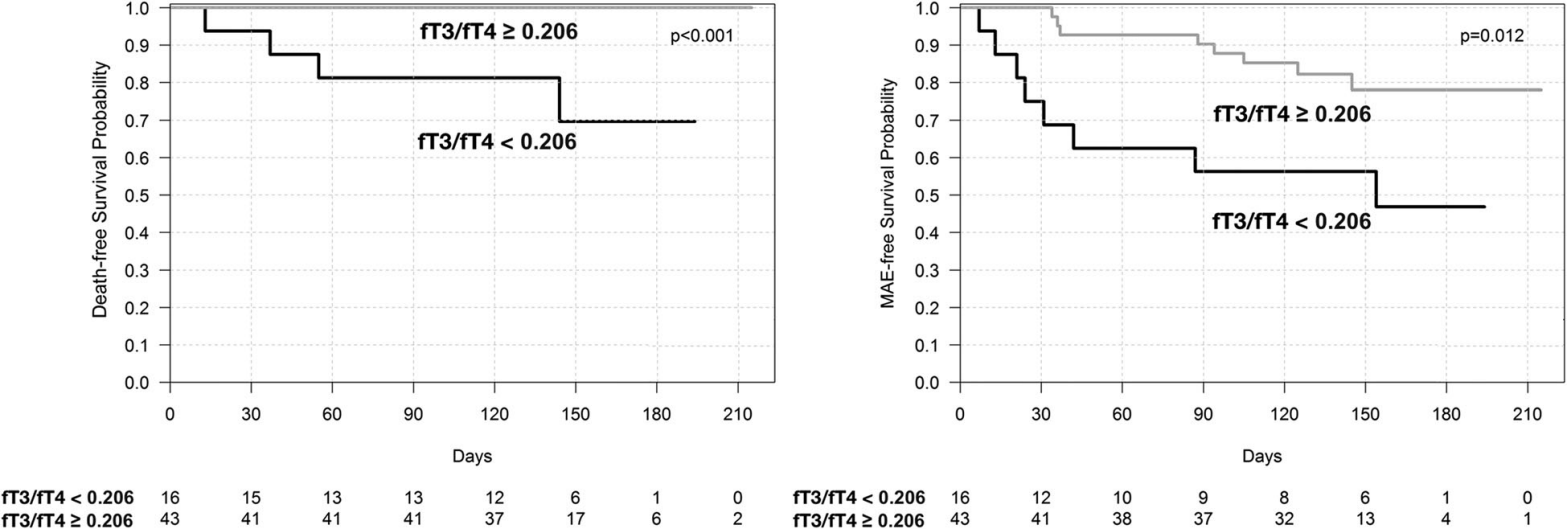
Kaplan-Meier survival curves for patients with low T3/fT4 ratio vs. normal fT3/fT4 ratio

Kaplan-Meier survival curves for cardiovascular death and MAE in patients with low fT3 vs. normal fT3 are shown in Fig. 1 and in patients with low fT3/fT4 ratio vs. normal fT3/fT4 ratio in Fig. 2. The cutoff point of fT3 was 3.1 pmol/l (laboratory norm). The optimal cutoff point of fT3/fT4 ratio (calculated by ROC curve analysis) was 0.206 for both cardiovascular mortality and MAE. The Kaplan-Meier survival curves indicate marked differences between the groups.

## Discussion

Our results show that low fT3 concentration is frequently found in severe HF patients (15.3%). Of note, as TSH and fT4 remained normal, low T3 may be missed if only routine TSH measurement is done. This seems to be an important finding, as Group A, in comparison to Group B, presented not only worse initial clinical findings, laboratory and echocardiographic parameters (pivotal in assessment of HF severity) but also an unfavorable HF course. The significance of fT3/fT4 ratio was also found by Kozdag et al., who proved that it is an independent prognostic marker in HF [27]. In our study, a univariate analysis revealed that the fT3 and fT3/fT4 ratio is associated with a poorer prognosis in HF. The Kaplan-Meier survival curves showed that the death-free survival probability and MAE-free survival probability are lower amongst patients with a lower fT3 and fT3/fT4 ratio. Our findings are consistent with previous research [20, 22–24, 27].

Low fT3 levels and fT3/fT4 ratio are associated with more advanced HF, reflected by echocardiographic variables. Another finding was a high incidence of selenium deficiency in our HF patients (74.6%). However, we could not prove correlation between selenium concentration and fT3 levels. Thus, our hypothesis of Se deficiency being a cause of impaired TH conversion could not be confirmed and the mechanism of low fT3 syndrome in HF patients remains controversial. Our study is the first to date testing the correlation between Se and fT3 levels in HF patients. The negative result is an argument against the hypothesized role of Se deficiency as an important contributor of low T3 syndrome. However, our study confirms a high incidence of selenium deficiency in HF patients and the importance of this phenomenon deserves further research.

The correlation between fT3 and hsCRP levels in our study may indicate that inflammation may contribute to impaired TH conversion. Our observations are consistent with Lubrano et al. [28].

Our study documents the importance of low fT3 levels being linked to HF severity and a poor prognosis. Interestingly, heart transplantation is associated with normalization of low T3 levels [21]. Low fT3 levels may be the result of severe multiorgan syndrome of HF. Future studies in larger groups are needed to evaluate the independent prognostic value of low T3. On the other hand, we cannot exclude the possibility that low T3 syndrome itself may worsen the HF course and if such a concept is proven, a low fT3 level may become a novel therapeutic target in HF. Few publications analyzed T3 supplementation in HF. Research by Hamilton et al. revealed that intravenous T3 supplementation in 23 selected patients with advanced HF and low T3 levels is well tolerated without episodes of ischemia or clinical arrhythmia and an increase in mean metabolic rate assessed by indirect calorimetry. T3 supplementation does not affect mean arterial blood pressure and heart rate. Nevertheless, it causes a significant increase in cardiac output with a reduction in systemic vascular resistance [29]. In addition, Pingitore et al. showed that intravenous T3 replacement therapy causes a decrease in heart rate, an increase in left ventricular diastolic volume and stroke volume, as well as an improvement of a neurohormonal profile: a decrease of plasma noradrenaline, NT-proBNP and aldosterone [30]. Amin et al. [31] proved that lowering of NT-proBNP and hsCRP levels may be accompanied by an increase in LVEF and 6-min walk distance. Another study conducted by Holmager et al. [32] concerning oral T3 supplementation did not support the hypothesis of T3 supplementation in chronic HF. However, in that study, the sample size was also small (only 13 participants completed the protocol) and not representative, so there is no definite answer as to whether such an approach may be beneficial and further studies are needed.

Our study has several strengths and limitations. One advantage is the prospective character of the protocol and very strict inclusion criteria. Unlike other cited studies, this approach allowed the creation of a very homogenous group with exclusion of subclinical hypothyroidism and many common factors potentially influencing the thyroid status. On the other hand, such narrow criteria made the study population relatively small and did not enable a significant multivariate analysis of the prognostic value of low fT3 levels.

## Conclusions

Low T3 syndrome is frequently found in patients with HFrEF and is associated with a poor outcome. Selenium deficiency is highly prevalent in severe HF patients, but we did not identify any significant correlation between selenium and fT3 levels. Further studies concerning the mechanisms and importance of low fT3 level in the heart failure population are needed.

## Abbreviations

+dp/dt: rate of left ventricle systolic pressure rise
AKT: serine/threonine-protein kinase
D1: deiodinase 1
D3: deiodinase 3
E/A index: E wave velocity to A wave velocity ratio
E/E’ index: E wave velocity to E’ wave velocity ratio
ECLIA: electrochemiluminescent immunoassay
fT3: free triiodothyronine
fT4: free tetraiodothyronine
Ge74: germanium isotope
GPX: glutathione peroxidase
HF: heart failure
hsCRP: high sensitivity C-reactive protein
ICP-MS: inductively coupled plasma mass spectrometry
LVEDD: left ventricle end-diastolic diameter
LVEDV: left ventricle end-diastolic volume
LVEF: left ventricle ejection fraction
LVESD: left ventricle end-systolic diameter
LVESV: left ventricle end-systolic volume
MAPSE: mitral annular plane systolic excursion
NT- proBNP: N-terminal pro B-type Natriuretic Peptide
PI3K: phosphatidylinositol 3-kinase
PLN: fosfolamban
ROS: reactive oxygen species
rT3: reverse triiodothyronine
Se: selenium
SERCa2: sarcoplasmic/endoplasmic reticulum calcium ATPase2
SI: sphericity index
T3: triiodothyronine
T4: tetraiodothyronine
TAPSE: tricuspid annular plane systolic excursion
TH: thyroid hormones
TSH: thyroid stimulating hormone
α-MHC: myosin heavy chain α
