# Correction to: Low triiodothyronine syndrome and selenium deficiency - undervalued players in advanced heart failure? A single center pilot study

**DOI:** 10.1186/s12872-019-1118-z

**Published:** 2019-06-03

**Authors:** Magdalena Fraczek-Jucha, Katarzyna Zbierska-Rubinkiewicz, Małgorzata Kabat, Krzysztof Plens, Radoslaw Rychlak, Jadwiga Nessler, Andrzej Gackowski

**Affiliations:** 1 Department of Coronary Disease and Heart Failure, Jagiellonian University, Medical College, Krakow, Poland; 2 Department of Emergency Medical Care, Jagiellonian University Medical College, Krakow, Poland; 3 Department of Coronary Disease and Heart Failure, John Paul II Hospital, Krakow, Poland; 4 Krakow Cardiovascular Research Institute, Krakow, Poland

**Correction to: BMC Cardiovasc Disord**

**https://doi.org/10.1186/s12872-019-1076-5**

After publication of the original article [1], the authors have notified us of a typing error in spelling Dr. Kabat’s name. The original publication has been corrected.

• Originally published name:
- Małgorzta Kabat
• Corrected name:
- Małgorzata Kabat
